# The Box-Cox power transformation on nursing sensitive indicators: Does it matter if structural effects are omitted during the estimation of the transformation parameter?

**DOI:** 10.1186/1471-2288-11-118

**Published:** 2011-08-19

**Authors:** Qingjiang Hou, Jonathan D Mahnken, Byron J Gajewski, Nancy Dunton

**Affiliations:** 1 Department of Biostatistics, University of Kansas Medical Center, Kansas City, KS 66160, USA; 2 Schools of Nursing and Allied Health, University of Kansas Medical Center, Kansas City, KS 66160, USA; 3 School of Nursing, University of Kansas Medical Center, Kansas City, KS 66160, USA

**Keywords:** Data transformation, NDNQI, Nursing quality indicator, ANOVA, Mixed model

## Abstract

**Background:**

Many nursing and health related research studies have continuous outcome measures that are inherently non-normal in distribution. The Box-Cox transformation provides a powerful tool for developing a parsimonious model for data representation and interpretation when the distribution of the dependent variable, or outcome measure, of interest deviates from the normal distribution. The objectives of this study was to contrast the effect of obtaining the Box-Cox power transformation parameter and subsequent analysis of variance with or without a priori knowledge of predictor variables under the classic linear or linear mixed model settings.

**Methods:**

Simulation data from a 3 × 4 factorial treatments design, along with the Patient Falls and Patient Injury Falls from the National Database of Nursing Quality Indicators (NDNQI^®^) for the 3^rd ^quarter of 2007 from a convenience sample of over one thousand US hospitals were analyzed. The effect of the nonlinear monotonic transformation was contrasted in two ways: a) estimating the transformation parameter along with factors with potential structural effects, and b) estimating the transformation parameter first and then conducting analysis of variance for the structural effect.

**Results:**

Linear model ANOVA with Monte Carlo simulation and mixed models with correlated error terms with NDNQI examples showed no substantial differences on statistical tests for structural effects if the factors with structural effects were omitted during the estimation of the transformation parameter.

**Conclusions:**

The Box-Cox power transformation can still be an effective tool for validating statistical inferences with large observational, cross-sectional, and hierarchical or repeated measure studies under the linear or the mixed model settings without prior knowledge of all the factors with potential structural effects.

## Background

Many health and nursing related studies focus on outcome measures that can be used to identify superior treatments and/or to reveal deficiencies in practices [1]. While substantial effort has been made on research design and data collection, researchers are more concerned with the validity of statistical conclusions should the reliability of the measurement be compromised [2] or the basic statistical assumptions be violated because non-normal data distributions with these outcomes are common [3]. In the later case, data transformation is one of the powerful tools for developing parsimonious models for detecting structural effects or predictive factors and for better data representation and interpretation [4-6]. Ever since the pioneer works on the formal estimation of a suitable transformation [3], the nonlinear monotonic power transformation family in the form of $y^{\left(\lambda \right)}=\frac{y^{\lambda }-1}{\lambda },\;if\;\left(\lambda \neq 0\right)$ and *y*^(*λ*) ^= log (*y*), *if*(*λ *= 0) has been the focus of extensive research and, as a result, has resulted in widespread applications in linear model analysis. With the advance in statistical research and computational technology, the Box-Cox transformation has recently found its application in the linear mixed model settings [7-9], which, as hierarchical experiment design and longitudinal studies become more desirable, is an active field of research. Under the linear model framework, the parameter estimate for λ with the power transformation family, by definition, is obtained along with the structural effect such that the error term is normally distributed, *ε *~ N (0, σ^2^), with the model ***y***^(*λ*) ^= **Xθ **+ ***ε***, where ***y***^(*λ*)^, **X**, and **θ **represents the transformed response, the design matrix of structural effects, and the vector of parameter estimates, respectively. This implies one should know a priori what the structure is before actually estimating the parameter for transformation (λ). In reality, factors with potential structural effects on the outcome can be large, unknown, and often are of primary interest for research, especially for large observational or cross-sectional studies, such as the National Database of Nursing Quality Indicators (NDNQI^®^). This study contrasted the effect of obtaining the Box-Cox power transformation parameter and subsequent analysis with or without a priori knowledge of predictor variable under the classic ANOVA model with simulation, and then illustrated such effects by extending the Box-Cox transformation into hierarchical analysis with the mixed model on two NDNQI nursing sensitive indicators.

### Basic assumption for linear model methodology

Statistical analyses with the linear model methodology are based on the assumption that the population being investigated is normally distributed with a common variance and additive mean structure [10,11]. Let *Y*_*ijk *_be the response for the k^th ^unit in the ij^th ^subclass for a two-way classification model; *β *is the vector of regression parameters, and *X*_*ij *_is the design matrix for the ij^th ^subclass, the linear model (1) then assumes that the error is independent and identically distributed normal variable, *ε*_*ijk *_~ *N *(0, σ ^2 ^), after removing the structural effect *X*_*ij*_*β*.

(1) $$Y_{ijk}=X_{ij}\beta +\varepsilon_{ijk}$$

When the theoretical assumption is not satisfied, data transformation can be applied so that inferences about unknown factors are still valid on the transformed scale [11]. Depending on the type of data and the form of their distribution, a number of different transformations were found so that the transformed data would meet the theoretical assumptions. These include: logit transformations for proportions; the square root transformation for count data; a logarithm or inverse transformation for continuous data skewed to either side with a heavy tail, etc. The family of power transformations is useful when the choice of transformation to improve the approximation of normality is not obvious [12]. The power transformation was first introduced by Tukey [13] and later modified by Box & Cox [3] to take account of the discontinuity at *λ *= 0. The Box-Cox power transformation takes the following form (2) so that the transformed values are a monotonic function of the observations,

(2) $$y_{i}^{\left(\lambda \right)}=\left\{\begin{array}{cc} \left(y_{i}^{\lambda }-1\right)∕\lambda ; & \lambda \neq 0\\ \log\left(y_{i}\right); & \lambda =0, \end{array}\right)$$

and for the unknown transformation parameter, *λ*,

(3) $$Y_{ijk}^{\left(\lambda \right)}=X_{ij}\beta +\varepsilon_{ijk}$$

where, *Y*_*ijk*_, *X*_*ij*_, *β *and *ε*_*ijk *_are all defined as in equation (1). This transformation may allow the response variable to achieve simplicity and additivity in mean structure for the expected value of (*y*^*λ*^) and make the variance more nearly constant among points in the factor space [14].

Substantial research has been conducted on the theoretical aspects of Box-Cox modification [15], and a wide variety of applications used Box-Cox transformation [16-18]. It is reported that maximum likelihood-based variance components analysis applied to non-normal data had inflated type I errors, which were controlled best by Box-Cox transformation [19]. Box-Cox transformation can be used to improve signal/noise ratio, map families of distributions and result in more efficient and robust results [20]. Analysis of the diagnostic accuracy using the receiver operating characteristic curve methodology required a Box-Cox transformation within each cluster to map the test outcomes to a common family of distributions [21]. Recently, median regression after applying the Box-Cox transformation was reported as notably more efficient and robust than the standard least absolute deviations estimator [22]. Due to its highly structured nature, however, the Box-Cox power transformation model is controversial, as some theoretical and Monte Carlo studies indicated that the data based estimate of *λ *is unstable and that, much like the case of multivariate collinearity, *λ *and *β *are highly correlated [7-9,16,17]. Other studies, however, downplayed the cost from data-based Box-Cox transformation, arguing the cost should be moderate on the whole and seldom large [23]. It has been suggested that we need to understand better the joint effects of variable selection and data transformation [7,8,23]. Under the Box-Cox transformation (2), one can put the data on the correct scale for an ANOVA model when the predictor variables (***X***) are identified and included during the transformation process. Unfortunately, for many non-randomized studies it is not clear what predictor variables should be included when the dependent variable deviates significantly from the normal distribution.

Under the linear mixed model setting, the error term of *ε*_*ijk *_in model (3) is no longer independent and identically distributed (iid) normal, but rather correlated because sampling and experiment units may be hierarchical or each sampling unit may be repeatedly measured.

### NDNQI database overview

In 1998, NDNQI^® ^was established by the American Nurses Association (ANA) to monitor nursing-sensitive indicators that measure nursing quality and patient safety across all 50 states in the US [24]. Over the last decade, NDNQI has seen its participating hospitals grow from 35 in 1998 up to 1,450 by the end of 2009 [25]. With nursing data collected at the unit level within member institutions, NDNQI provides hospitals unit-level performance reports with 8-quarter trend data, along with national comparison data grouped by hospital staffed bed size, teaching status, Magnet status, various other hospital characteristics, and unit type [25].

Nursing-sensitive indicators reflect the structure, process and outcomes of nursing care. Examples of nursing structure measures include the supply of nurses, skill level, RN education and certification [24-26]. The Patient Falls indicator is an example of a nursing sensitive outcome and is defined as the rate per 1,000 patient days at which patients experience an unplanned descent to the floor during the course of their hospital stay.

$$\frac{Total\;Number\;of\;Patient\;Falls\times 1,000}{Total\;Number\;of\;Patients\;Days}$$

Patient Injury Falls, as another example, is defined as:

$$\frac{Total\;Number\;of\;Patient\;Falls\;Leading\;to\;Injury\times 1,000}{Total\;Number\;of\;Patient\;Days}$$

Both Patient Falls and Patient Injury Falls have a common denominator of Total Number of Patient Days. Conceptually, a patient day is 24 hours, beginning with the hour of admission. The operational definition of patient days is the total number of inpatients present at the midnight census plus the total number of hours of short stay patients divided by 24. Short stay patients are patients on a unit for less than 24 hours either for observation or same day surgery.

Both Patient Falls and Patient Injury Falls are critical nursing quality indicators that may be associated with nursing workforce characteristics, as well as with unit type and some hospital characteristics such as teaching status and Magnet status. Other unknown factors might also affect the rates of Patient Falls and Patient Injury Falls in NDNQI hospitals across a wide spectrum of settings over the entire United States. Further, if such factors do exist, it would be of great interest to examine what administrative or nursing process adjustments a hospital might take to reduce these rates and thus improve the overall quality of service.

## Methods

The Box-Cox power transformation requires all predictor variables to be included in the model for estimating transformation parameter in order to put a skewed response onto the correct scale for the classic ANOVA model [27]. In this paper, a Monte Carlo simulation with a 3 × 4 factorial treatment design was used to contrast the properties of power-transformed response variables with and without the presence of the 3 × 4 factorial structural effects when the transformation parameter was estimated. The residual and the treatment main effects with the simulation were examined with two-way ANOVA model. NDNQI Patient Falls and Patient Injury Falls, collected on unit level, are correlated within hospitals and right-skewed in distribution. Statistical analysis without data transformation may violate the underlying assumption because of non-normal error distributions, potentially also compounded with a correlated covariance structure. For illustration purpose, we first ignored the within hospital intra class correlation (ICC) and then extended the Box-Cox power transformation into the linear mixed model framework [26] and analyzed NDNQI Patient Falls and Patient Injury Falls with mixed models assuming compound symmetric covariance structure [28] to contrast the effect of Box-Cox transformations when predictor variable (Hospital Teaching and Magnet Status) were included in the transformation model with when they were ignored. Note, in NDNQI quarterly reports, ICC for all indicators were actually properly adjusted [29].

Patient Falls and Patient Injury Falls data from 6726 nursing units in 926 hospitals for the 3^rd ^quarter in 2007 were extracted from the NDNQI database maintained by NDNQI project at The Kansas University School of Nursing. The number of nursing units per hospital ranged from 1 to 36 with a median of 6 ± 5 (interquartile range). Along with the two indicators, hospital teaching status (Academic Medical Center; Other Teaching; Non-Teaching) and Magnet status (Magnet vs. Non-Magnet) were chosen from a variety of stratification variables for illustrative purposes. Box-Cox transformation on Patient Falls and Patient Injury Falls were then applied both with and without inclusion of these predictors in the model with which the power transformation parameters were estimated.

### Monte Carlo Simulations

All simulated data are based on a completely randomized block design with 3 × 4 factorial treatments, which can be expressed in the following model

(4) $${ϒ}_{ijk}^{\left(\lambda \right)}=\alpha_{i}+\beta_{j}+\gamma_{ij}+\varepsilon_{ijk}$$

where ${ϒ}_{ijk}^{\left(\lambda \right)}$ represented the transformed response from the *k*^th ^block with the *i*^th ^treatment for factor A and *j*^th ^treatment for factor B; *μ *was the overall mean; *α*_*i *_was the *i*^th ^treatment effect for factor A; *β*_*j *_was the *j*^th ^treatment effect for factor B, *γ*_*ij *_represented the factor A, B interaction, and *ε*_*ijk *_~ *N *(0, σ^2^) represents error terms that followed the normal distribution. The transformed response vector ${ϒ}_{ijk}^{\left(\lambda \right)}$ in (4) was generated as the sum of the two factor main effects plus their interaction with *α*_1 _**= **3.6; *α*_2 _**= **4.5; *α*_3 _**= **5.4; *β*_1 _= 2.0; *β*_2 _= 2.4; *β*_3 _= 2.8; *β*_4 _= 3.2; and *γ*_*ij *_= *α*_*i *_× *β*_*j *_for *i *= 1, 2, 3 and *j *= 1, 2, 3, 4; respectively. The random error *ε*_*ijk *_was generated as *N *(0, 26). The non-transformed response vector was then obtained through the inverse of power transformation function (2)Y_*ijk *_= (${ϒ}_{ijk}^{\left(\lambda \right)}$ * *λ *+1)^(1/λ) ^with the power transformation parameter (*λ*) being fixed at 0.4. Parameter *α*_*i*_, *β*_*j *_and *ε*_*ijk *_in model (4) were set such that the main effect and their interaction were all important. To check for large sample properties we let the replication for each combination of factors vary from 4 to 24 by 2, corresponding to the sample size ranges from 48 to 288 by 24. Two estimated power transformation parameters were obtained for each simulated data set: the first with the 3 × 4 factorial effect included as predictor variables in the transformation model (*λ*_*1*_), representing the Box-Cox transformation by definition; and the other just a power transformation of the response variable (*λ*_*0*_), representing an approximation one might see in practice. Both power transformed response variables ${\left({ϒ}_{ijk}^{\left(\lambda \right)}\right)}^{\left(\lambda_{1}\right)}$ and ${\left({ϒ}_{ijk}^{\left(\lambda \right)}\right)}^{\left(\lambda_{0}\right)}$ were then used as the dependent variables for separate ANOVAs with 3 × 4 factorial treatment effects. The *F *statistics and *P*-values for the two factor main effects along with their interaction effects from the ANOVA tables were compared under the different power transformations. Residuals after the main effects and their interaction for both models were examined for normality with the Shapiro-Wilk statistic. The power transformation parameter was obtained following the maximum likelihood method [3]. A total of 1000 simulated data sets were generated for each set of replicate ranging from 4 to 24 for a completely randomized block design with 3 × 4 factorial treatments. SAS, version 9.2 was used for data generation and statistical analyses [30].

The simulation study on a two factor, completely randomized, block design was aimed to answer the following two questions.

1. Will the goal of simplicity in structure and homogeneity in error for transformation be still achievable if predictor variables are omitted from the power transformation model?

2. What are the consequences of conducting the analysis of variance on the transformed response variable without including the predictor variables in estimating the transformation parameter (*λ*)?

### Application to NDNQI Indicators

Suppose one is interested in investigating Patient Falls or Patient Injury Falls as a function of hospital teaching and/or Magnet status, then X_*ij *_in (1) has 6 columns with the first being a column of 1's, the 2^nd ^and 3^rd ^representing the teaching status, the 4^th ^an indicator for Magnet status, and the 5^th ^and 6^th ^for the Teaching by Magnet status interaction. After exploratory data analysis using the ANOVA model with hospital teaching and Magnet status as having structural effects, Patient Falls and Patient Injury Falls were analyzed with the mixed model under a) without transformation, b) power transformed without teaching and Magnet effects during the parameter estimation for (λ_0_), and c) power transformed with teaching and Magnet effects during the parameter estimation (λ_1_). The power transformation parameter, λ_0_, was obtained through a grid search by maximizing the log likelihood of the residual for the transformed response variable after removing the overall means. As Gurka et al. [7] proposed, we obtained λ_1_through maximizing the residual maximum likelihood (REML) with the existing computational procedures (SAS PROC Mixed). Specifically, for each indicator, a scaled Box-Cox transformation [3] for a wide range of the power parameter value, λ_*i *_(*i *= 1 to 8 by 0.01) was first applied. Then, each transformed response was analyzed with the compound symmetry covariance structure to model the correlation among units within hospital. The λ_*i *_that corresponds to the maximum REML was selected as λ_0_.

## Results

**Figure 1:**
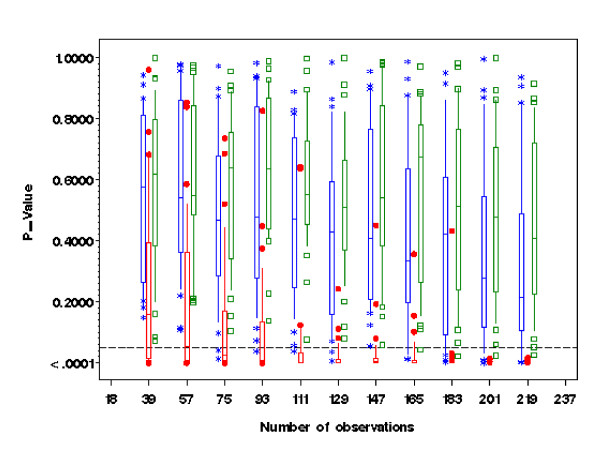
**Test for normality by Shapiro-Wilk statistic for residual obtained from 3 × 4 ANOVA with response variable being non-transformed (red), power transformed without treatment effect (blue), and power transformed with treatment effect and their interaction (green)**. The horizontal dot line represents 5% significant level. All box plots are obtained from 30 datasets selected at random from 1000 simulation (for clarity) with whiskers representing the 10^th ^and 90^th ^percentiles.

One of the main objectives for the Box-Cox power transformation is to achieve normality in random error distribution after removing the additive effects. With simulated data under model (4), residuals from the 3 × 4 factorial ANOVA models with either ${\left({ϒ}_{ijk}^{\left(\lambda \right)}\right)}^{\left(\lambda_{1}\right)}$ or ${\left({ϒ}_{ijk}^{\left(\lambda \right)}\right)}^{\left(\lambda_{0}\right)}$as response variable showed limited evidence of non-normality across a wide range of sample size as assessed with the Shapiro-Wilk test (Figure 1). Conversely, the corresponding normality tests for residuals with non-transformed response variable ${ϒ}_{ijk}^{\left(\lambda \right)}$ were all significant at the 5% level. The Box-Cox power-transformed response led to (approximate) normality in the distributions of residuals after removing the additive effects of model (1). The inclusion or exclusion of the predictor variables in the transformation model made little difference in terms of normality for residual distribution. Table 1 shows the empirical mean and standard deviation for *λ*_*1 *_and *λ*_*0 *_along with preset transformation parameter *λ*. For each set of replicates, normality tests for residual are mostly non-significant (> 80%) with either ${ϒ}_{ijk}^{\left(\lambda_{1}\right)}$ or ${ϒ}_{ijk}^{\left(\lambda_{0}\right)}$ as the dependent variable, but with the non-transformed response Y_*ijk *_the majority of distributions of residuals were significantly different from the normal distribution, especially when the number of replicates was high.

**Table 1:** Statistics for power transformation parameter and statistical test for structural effects based on Monte Carlo simulations

Simulations with non negative estimate (*λ*)	Sample size	Transformation parameter and its empirical estimate ± Standard deviation			Test for residual normality (proportion with P > 0.05)		
N	n	*λ*	*Mean *(*λ*_*1*_) ± *Std*	*Mean *(*λ*_*0*_) ± *Std*	*Y*^*λ*^	${\left(Y^{\lambda }\right)}^{\left(\lambda_{0)}\right)}$	${\left(Y^{\lambda }\right)}^{\left(\lambda_{1}\right)}$

973	36	0.4	0.397 ± 0.160	0.296 ± 0.123	0.698	0.962	0.968
996	54	0.4	0.386 ± 0.125	0.289 ± 0.107	0.522	0.950	0.971
999	72	0.4	0.393 ± 0.104	0.295 ± 0.010	0.387	0.963	0.978
1000	90	0.4	0.395 ± 0.089	0.295 ± 0.087	0.251	0.941	0.977
1000	108	0.4	0.393 ± 0.085	0.295 ± 0.083	0.179	0.929	0.975
1000	126	0.4	0.393 ± 0.075	0.296 ± 0.074	0.120	0.920	0.976
1000	144	0.4	0.395 ± 0.068	0.299 ± 0.067	0.082	0.912	0.979
1000	162	0.4	0.395 ± 0.067	0.398 ± 0.068	0.056	0.891	0.972
1000	180	0.4	0.395 ± 0.063	0.030 ± 0.063	0.037	0.889	0.968
1000	198	0.4	0.396 ± 0.059	0.301 ± 0.059	0.018	0.880	0.971
1000	216	0.4	0.396 ± 0.057	0.302 ± 0.057	0.010	0.867	0.977

Notation: *λ *is the preset value for generating the data; *λ*_*0 *_represents the estimated value for the transformation parameter with no factorial treatment effect in the model; *λ*_*1 *_stands for estimated value of transformation parameter with factorial treatment effect in the model. P-value is obtained by Shapiro-Wilk test with SAS Univariate procedure. A total of 1000 datasets were generated for each fixed sample size and transformation parameter.

**Figure 2:**
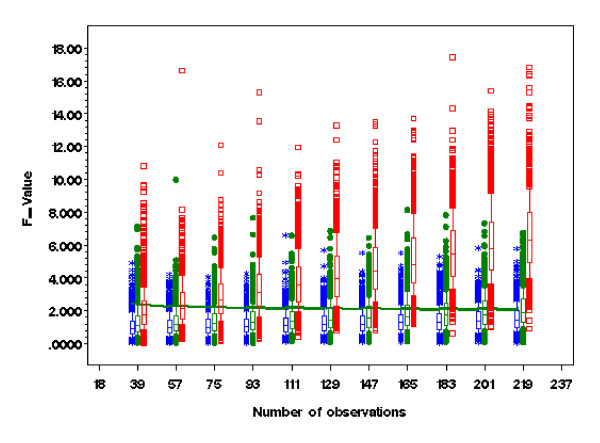
**F-values from ANOVA table for the interaction effect by 3 × 4 factorial treatment design on response variable being non-transformed (red), power transformed without treatment effect (blue), and power transformed with treatment effect and their interaction (green)**. The green line represents 5% significant level. All box plots are obtained from 1000 simulated datasets with whiskers representing the 10^th ^and 90^th ^percentiles.

The other objective with Box-Cox power transformation is to achieve simplicity and additivity by strengthening the main effects while reducing the effect of interaction terms [3]. In regard to the two factor main effect, the same conclusion was reached with either ${\left({ϒ}_{ijk}^{\left(\lambda \right)}\right)}^{\left(\lambda_{1}\right)}$ or ${\left({ϒ}_{ijk}^{\left(\lambda \right)}\right)}^{\left(\lambda_{0}\right)}$ as the response variable, as reflected by the respective linear model analyses. Both transformations strengthened the main effect through reducing the effect of interaction, as illustrated by Box & Cox [3] in their example data set. Figure 2 contrasts the two different power transformations along with non-transformed data on interaction effects. Without transformation, most of the two factor interaction effects were significant (P < 0.05). Interestingly, either transformation of the response variable alone or with the predictor variables in the model tended to reduce the interaction effects towards a non-significant level (P > 0.05). Empirical means and standard deviations for the F-values and significance test for interaction effects through simulation revealed the same trend for a wide range of sample sizes (Table 2). For fixed *β *in (2), the larger the variance *σ*^2^, the closer the estimate of λ_1 _and λ_0 _to the prefixed power transformation parameter λ. With λ = 0.4, λ_1 _was always larger than λ_0 _(Table 1). F-values for the factorial treatment effects tended to be slightly higher with ${ϒ}_{ijk}^{\left(\lambda_{1}\right)}$ as the dependent variable than the corresponding effects for ${ϒ}_{ijk}^{\left(\lambda_{0}\right)}$, making the test for main effects a bit more conservative, and thus reducing the chance for committing type I error based on ${ϒ}_{ijk}^{\left(\lambda_{0}\right)}$.

**Table 2:** Statistics for tests of structural effect with different transformation models based on Monte Carlo simulations

Simulations with non negative estimate (*λ*)	Sample size	F-value for interaction effects ± STD with different model for power transformation			F-test for interaction effects (Proportion with P > 0.05)		
N	n	*MeanF*_*λ *_± *Std*	*MeanF*_*λ0 *_± *Std*	*MeanF*_*λ1 *_± *Std*	*Y*^*λ*^	${\left(Y^{\lambda }\right)}^{\left(\lambda_{0)}\right)}$	${\left(Y^{\lambda }\right)}^{\left(\lambda_{1}\right)}$

973	36	2.072 ± 1.372	1.083 ± 0.699	1.328 ± 0.922	0.687	0.885	0.938
996	54	2.460 ± 1.407	1.117 ± 0.673	1.338 ± 0.868	0.523	0.874	0.937
999	72	2.866 ± 1.475	1.126 ± 0.660	1.378 ± 0.836	0.375	0.854	0.935
1000	90	3.264 ± 1.656	1.178 ± 0.649	1.471 ± 0.865	0.343	0.823	0.927
1000	108	3.796 ± 1.686	1.219 ± 0.700	1.537 ± 0.908	0.158	0.802	0.903
1000	126	4.276 ± 1.900	1.292 ± 0.696	1.656 ± 0.951	0.100	0.748	0.899
1000	144	4.707 ± 1.984	1.336 ± 0.738	1.721 ± 0.993	0.066	0.722	0.866
1000	162	5.211 ± 2.156	1.411 ± 0.778	1.841 ± 1.073	0.043	0.697	0.852
1000	180	5.653 ± 2.080	1.444 ± 0.783	1.903 ± 1.053	0.016	0.663	0.834
1000	198	6.117 ± 2.282	1.497 ± 0.776	2.001 ± 1.114	0.009	0.624	0.798
1000	216	6.616 ± 2.324	1.570 ± 0.797	2.107 ± 1.114	0.003	0.589	0.788

Notation: *λ *is the preset value for generating the data; *λ*_*0 *_represents the estimated value for transformation parameter with no factorial treatment effect in the model; *λ*_*1 *_stands for estimated value of transformation parameter with factorial treatment effect in the model. P-value is obtained from ANOVA with SAS GLM procedure. A total of 1000 datasets were generated for each fixed sample size and transformation parameter.

**Figures 3:**
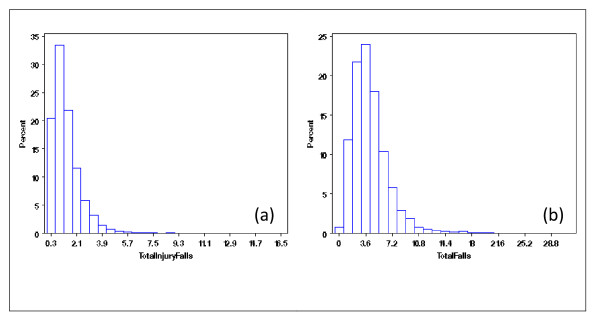
**Distribution of Total Falls (a) and Total Injury Falls (b), for NDNQI hospitals reported for 3^rd ^quarter, 2007**.

**Figure 4:**
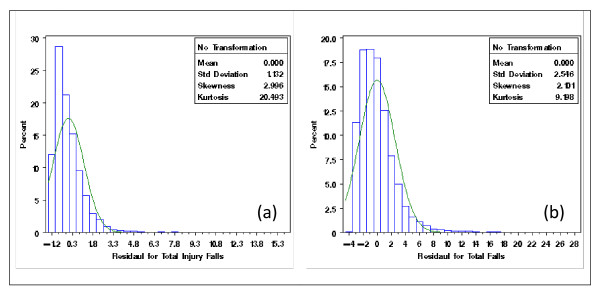
**Residual distribution (without data transformation) of Total Falls (a) and Total Injury Falls (b), for NDNQI hospitals reported for 3^rd ^quarter, 2007**.

**Figure 5:**
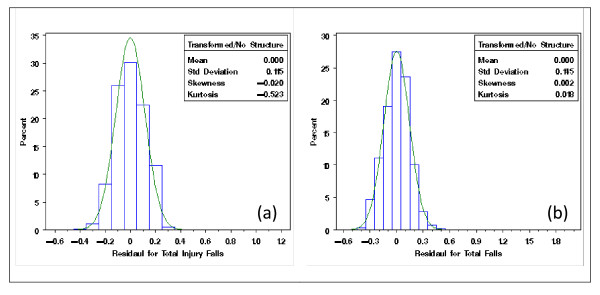
**Residual distribution of Total Injury Falls (a) and Total Falls (b), for NDNQI hospitals reported for 3rd quarter, 2007**. Residuals were obtained after removing the structural effect on power transformed dependent variable by hospital teaching and Magnet status. Here, the Box-Cox power transformation parameters were obtained with structural effects in the model.

**Figure 6:**
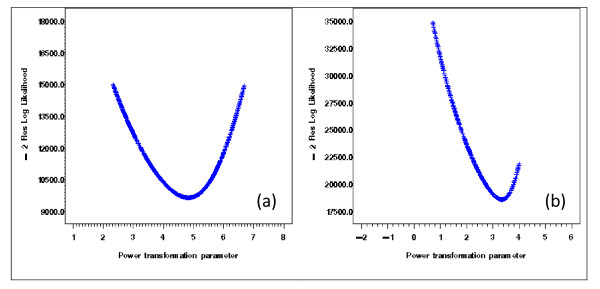
**Grid search for optimum Box-Cox power transformation parameters**. Residual Maximum Likelihood (REML) reached maxima at 3.34 and 4.82 for the Box-Cox power transformation parameters for Total Falls (a) and Total Injury Falls (b) estimated from repeated measure analysis with the linear mixed models.

**Figure 7:**
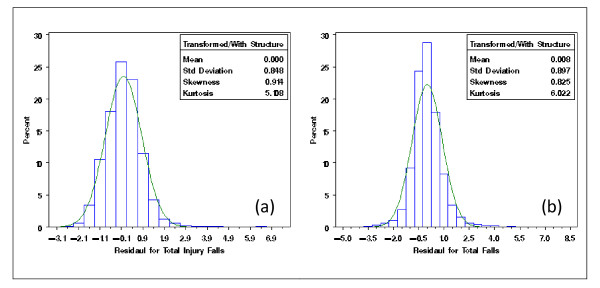
**Residual distribution of Total Injury Falls (a) and Total Falls (b), for NDNQI hospitals reported for 3^rd ^quarter, 2007**. Residual is obtained after removing the structural effect on power transformed dependent variable by hospital teaching and Magnet status. Here, the power transformation parameters were obtained without structural effect in the model.

**Figure 8:**
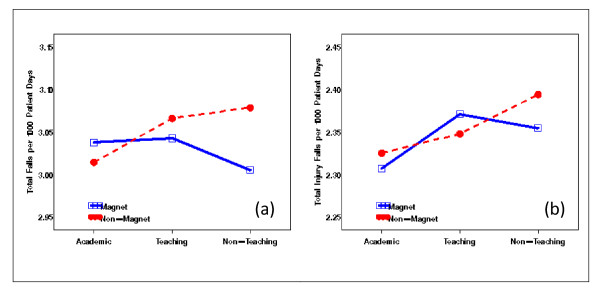
**Repeated measure analysis for structural effect by Magnet and teaching status for Patient Falls (a) and Patient Injury Falls (b) for NDNQI hospitals reported for 3^rd ^quarter, 2007**.

With the extracted NDNQI data, exploratory data analysis showed severely skewed distributions for Patient Falls and Patient Injury Falls (Figure 3a, b). Without transformation, the residuals after removing the structural effects of interest (teaching and Magnet status and their interaction) using mixed models differed clearly from normal distribution (Figure 4a, b). Residual distributions from the mixed model analyses with the 3 × 2 structural effects for hospital Teaching status and Magnet status for the transformed response ${ϒ}_{ijk}^{\left(\lambda_{0}\right)}$, obtained without removing the structural effects estimated (0.18 and -0.20, for Total Falls and Total injury Falls, respectively) are shown in Figures 5a &5b. With power transformation parameters λ_1 _(3.34 and 4.82, for Total Falls and Total injury Falls, respectively) (Figures 6a, b), residual distributions for Total Falls and Total injury Falls (Figure 7a, b) displayed similar patterns for the transformed response ${ϒ}_{ijk}^{\left(\lambda_{1}\right)}$ after removing the structural effects as for the transformed response ${ϒ}_{ijk}^{\left(\lambda_{0}\right)}$ without removing the structural effects (Figures 5a, b). The same conclusion can be reached by either transformation (Table 3). Hospitals with Magnet status generally had lower in Total Fall rates and Total Injury Fall rates than those without Magnet status. Hospitals without Magnet status were more likely to have higher Total Fall and Total Injury Fall rates if the hospital did not have a teaching function. Total Falls and Total Injury Falls for hospitals with Magnet status were less affected by their teaching status (Figures 8a, b).

**Table 3:** Repeated measure analysis with the linear mixed model for Patient Falls and Patient Injury Falls for 2007 NDNQI 3^rd ^quarter

Source of Variation distribution	Degree of Freedom	F-Value	Prob > F	Residual goodness-of-fit test for normal
Indicator: Total Falls (transformation with additive effect)				
Teaching Status	2	2.36	0.0945	
Magnet Status	1	3.83	0.0505	
Teaching × Magnet	2	5.15	0.0058	0.071

Indicator: Total Falls (transformation without additive effect)				
Teaching Status	2	2.73	0.065	
Magnet Status	1	5.91	0.0151	
Teaching × Magnet	2	7.14	0.0008	0.023

Indicator: Total Falls (no transformation)				
Teaching Status	2	2.14	0.1178	
Magnet Status	1	5.90	0.0151	
Teaching × Magnet	2	6.94	0.001	0.097

Indicator: Total Injury Falls (transformation with additive effect)				
Teaching Status	2	1.83	0.1603	
Magnet Status	1	9.37	0.0022	
Teaching × Magnet	2	4.14	0.016	0.117

Indicator: Total Injury Falls (transformation without additive effect)				
Teaching Status	2	1.37	0.2536	
Magnet Status	1	9.78	0.0018	
Teaching × Magnet	2	4.45	0.0118	0.029

Indicator: Total Injury Falls (no transformation)				
Teaching Status	2	6.55	0.0014	
Magnet Status	1	3.63	0.0569	
Teaching × Magnet	2	3.29	0.0373	0.146

Repeated measure analysis for Patient Falls and Patient Injury Falls for a) No transformation, b) transformed without structural effects), and c) transformed with structural effects. Residual goodness-of-fit tests for normal distribution is based on Kolmogorov-Smirnov statistic.

## Discussion

The Box-Cox power transformation provides an effective tool to justify the use of the linear model when the response variable is not normally distributed. It was originally defined as highly structured and required all predictor variables to be included in the power transformation model [3]. There is always a cost resulting from selection of the transformation expressed as an inflated variance [7,16]. However, predictor variables may not always be clearly defined in practice. This is especially true for exploratory data analysis, observational studies, or classification and regression tree (CART) analysis aimed at finding potential relationships when the distribution of the response variable deviates significantly from normality. In such cases, applying the Box-Cox power transformation to the response variable alone and then searching for potential predictor variables was demonstrated to be effective in terms of achieving constant error and simplicity of main effects in the simulations and examples we examined. In our simulated data, the statistical tests for main effects were slightly more conservative for ${\left({ϒ}_{ijk}^{\left(\lambda \right)}\right)}^{\left(\lambda_{0}\right)}$ as compared to ${\left({ϒ}_{ijk}^{\left(\lambda \right)}\right)}^{\left(\lambda_{1}\right)}$, while the residuals after removing the structural treatment effects were unlikely to deviate from normality in either case (Table 1). On the other hand, interaction effects with ${\left({ϒ}_{ijk}^{\left(\lambda \right)}\right)}^{\left(\lambda_{0}\right)}$ were generally less likely to be detected compared to ${\left({ϒ}_{ijk}^{\left(\lambda \right)}\right)}^{\left(\lambda_{1}\right)}$ as the response variable.

The real case examples with Patient Falls and Patient Injury Falls from the NDNQI database showed Box-Cox power transformations both with and without structural effects for teaching and Magnet status included in the models for estimating the transformation parameters were equally effective in normalizing the residual distributions (Figures 5a, b, 7a, b). Table 3 shows the test statistics from hierarchical analysis allowing for correlation between error terms for structural effect by stratification variables (Teaching, Magnet, and their interactions).

With over 1800 hospitals (one in every thee general hospitals in the U.S.) contributing nursing indicator data to the NDNQI database today, it is as critical to provide users with valid national comparative data in nursing-sensitive quality indicators. As hospitals are striving to improve the quality of their nursing service, they can turn to the NDNQI quarterly reports to identify potential problems. While most of the nursing quality indicators are skewed in distribution, the structural effects of hospital characteristics are not always clear. In such cases, the classic Box-Cox power transformation can be applied to the nursing quality indicators, for a specific category of unit (such as pediatric or post surgical) with linear model analysis, or all units within hospital under the mixed model framework, prior to identifying the structural effects from a potentially large pool of variables.

Both the simulation study and real case analysis with NDNQI quarterly report data demonstrated that the consequence of omitting a structural effect from the Box-Cox power transformation was limited. This is important given the fact that for many large health-related observational studies the number of potential structural effects may be quite large. As of 2008, NDNQI had over 20 potential structural effects for 34 nursing indicators. Participating hospitals benefit from meaningful, valid comparative information based on a number of demographic, social, administrative, and service related factors. Estimating Box-Cox power transformation parameters on indicators without including the unknown, or sometimes unmeasured, structural effects can still provide participating hospitals with statistically valid comparisons.

A few limitations need to be noted. First, the Box-Cox transformation works better only if the measure of interest relatively smoothly spread out. In other words, the method may fail if the data cluster on a few values. Secondly, it is necessary to conduct a grid search of the transformation in order to find the optimum parameter that maximizes the residual likelihood both under the linear and the mixed model settings. Otherwise, the subsequent analysis may differ depending on whether or not the structural effects were included in the estimating process for the transformation parameters. Our results suggested a fine grid search for the transformation parameter should be used regardless the inclusion of factors with potential structural effects and regardless of whether the analysis uses the linear or mixed model settings, because the agreement on test for the structural effects occurs only if both transformations are optimized. Lastly, potential interactions between parameter estimates for transformation and for linear and/or random effects remains unclear, and, interpretation for the transformed data analysis, as always, remains a challenge that warrants further research.

## Conclusions

The validity of linear mixed modeling via maximum likelihood relies on the underlying assumption that the random effects and residuals of the dependent variable are normally distributed. Many health and nursing related outcome measures deviate from this assumption. While at the same time, factors with potential structural effects are of major interest and yet to be identified. Therefore, the Box-Cox power transformation provides a powerful tool for developing parsimonious models (i.e. applying linear mixed modeling) for data representation and interpretation. By extending the power transformation into linear mixed model setting with NDNQI examples, we found limited difference from subsequent test of structural effects regardless of whether such structure is included or omitted during the parameter estimation for transformation. This allows analysts to transform variables earlier in the model building, making the process of applying Box-Cox transformation much easier in practice.

Future work would be to employ some sort of a latent class analysis [30] on the NDNQI data and look for structural relationships within each class.

## Abbreviations

NDNQI: National Database of Nursing Quality Indicators; ANOVA: Analysis of Variance; ICC: Intra Class Correlation; iid: independent and identically distributed; ANA: American Nurses Association.

## Competing interests

The authors declare that they have no competing interests.

## Authors' contributions

QH reviewed literatures, conducted statistical analysis, and drafted the manuscript; JM and BG advised on and supervised statistical analysis and provided critical input in drafting and revising the manuscript; ND supervised NDNQI data collection, evaluated unit-specific nurse-sensitive data, and provided overall guidance for the manuscript. All authors read and approved the final manuscript.

## Pre-publication history

The pre-publication history for this paper can be accessed here:

http://www.biomedcentral.com/1471-2288/11/118/prepub
